# Suspected upadacitinib-associated drug hypersensitivity mimicking disease exacerbation in ulcerative colitis

**DOI:** 10.1007/s12328-026-02342-z

**Published:** 2026-04-27

**Authors:** Akira Madarame, Ryoya Kanda, Yasuhiro Shimizu, Takao Itoi

**Affiliations:** 1 Department of Gastroenterology and Hepatology, Niizashiki Central General Hospital, 1-7-2 Tohoku, Niiza City, Saitama 352-0001 Japan; 2 Department of Gastroenterology and Hepatology, Tokyo Medical University Hospital, 6-7-1, Nishi-shinjuku, Shinjuku-ku, Tokyo, 160-0023 Japan

**Keywords:** Ulcerative colitis, Drug hypersensitivity reactions, Janus kinase inhibitors, Upadacitinib

## Abstract

A woman in her 40s with a 4-year history of pancolitis-type ulcerative colitis (UC) was admitted with acute severe colitis unresponsive to intravenous corticosteroids. Remission was achieved with infliximab, but maintenance therapy was switched to high-dose 5-aminosalicylic acid (5-ASA) because of infliximab-induced alopecia. Two months later, she experienced a moderate relapse while on 5-ASA, and filgotinib was administered. However, her symptoms worsened, necessitating a switch to upadacitinib. Despite treatment, diarrhea and increased C-reactive protein levels persisted. Contrast-enhanced computed tomography revealed diffuse colonic inflammation, and the withdrawal of 5-ASA failed to improve her condition. With upadacitinib deemed ineffective, she was hospitalized for tacrolimus induction. Upon admission, colonoscopy demonstrated active mucosal inflammation, and stool studies excluded infection. Tacrolimus induced rapid clinical remission within 10 days. A drug-induced lymphocyte stimulation test (DLST) revealed a stimulation index of 230% for upadacitinib. Considering the clinical deterioration during upadacitinib therapy, improvement after its discontinuation, and DLST positivity, the clinical course was compatible with a suspected drug hypersensitivity to upadacitinib. This case suggests that suspected drug hypersensitivity to Janus kinase inhibitors may mimic apparent disease exacerbation in UC and should be considered when clinical deterioration persists during therapy.

**Supplementary Information:**

The online version contains supplementary material available at 10.1007/s12328-026-02342-z.

## Introduction

The Janus kinase-signal transducer and activator of transcription (JAK–STAT) pathway is an intracellular signaling pathway for multiple proinflammatory signals and a significant therapeutic target in ulcerative colitis (UC) [1]. Upadacitinib, a selective JAK1 inhibitor, is approved as an induction therapy for moderate-to-severe UC. However, approximately 1–2% of patients experience worsening colitis [2].

Drug hypersensitivity reactions (DHRs) are a diverse group of immune-mediated reactions that occur after drug exposure [3]. According to the Gell and Coombs classification system, allergic reactions to drugs are categorized from type Ⅰ to Ⅳ, based on the predominant immune mechanisms [4]. Several cases of JAK inhibitor-associated DHRs have been reported, the majority of which present with cutaneous manifestations [5–8].

We report a case of suspected upadacitinib-induced DHR in a patient with UC who was successfully treated with tacrolimus, presenting with colitis symptoms and no rash. Informed consent was obtained for inclusion in this report.

## Case report

The patient—a woman in her 40s with a 4-year history of pancolitis-type UC—had poor adherence to oral medication and took 5-aminosalicylic acid (5-ASA) irregularly. Her medical history was unremarkable. She was referred to our hospital owing to worsening UC and was admitted for acute severe UC.

She was unresponsive to intravenous corticosteroids but achieved remission with infliximab and was subsequently discharged. The 5-ASA dose was increased to 3600 mg/day for remission maintenance. Although continued infliximab therapy was planned, the patient requested discontinuation because of hair loss.

**Fig. 1:**
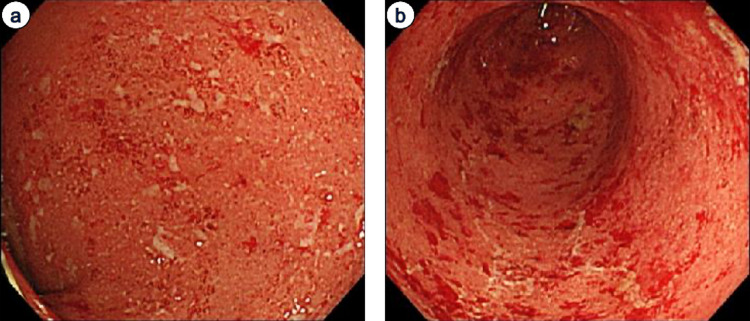
Sigmoidoscopy during relapse after infliximab discontinuation. **a**, **b** Sigmoidoscopy demonstrated continuous, diffusely distributed fine-granular mucosa with scattered erosions (**a** sigmoid colon, **b** rectum)

**Fig. 2:**
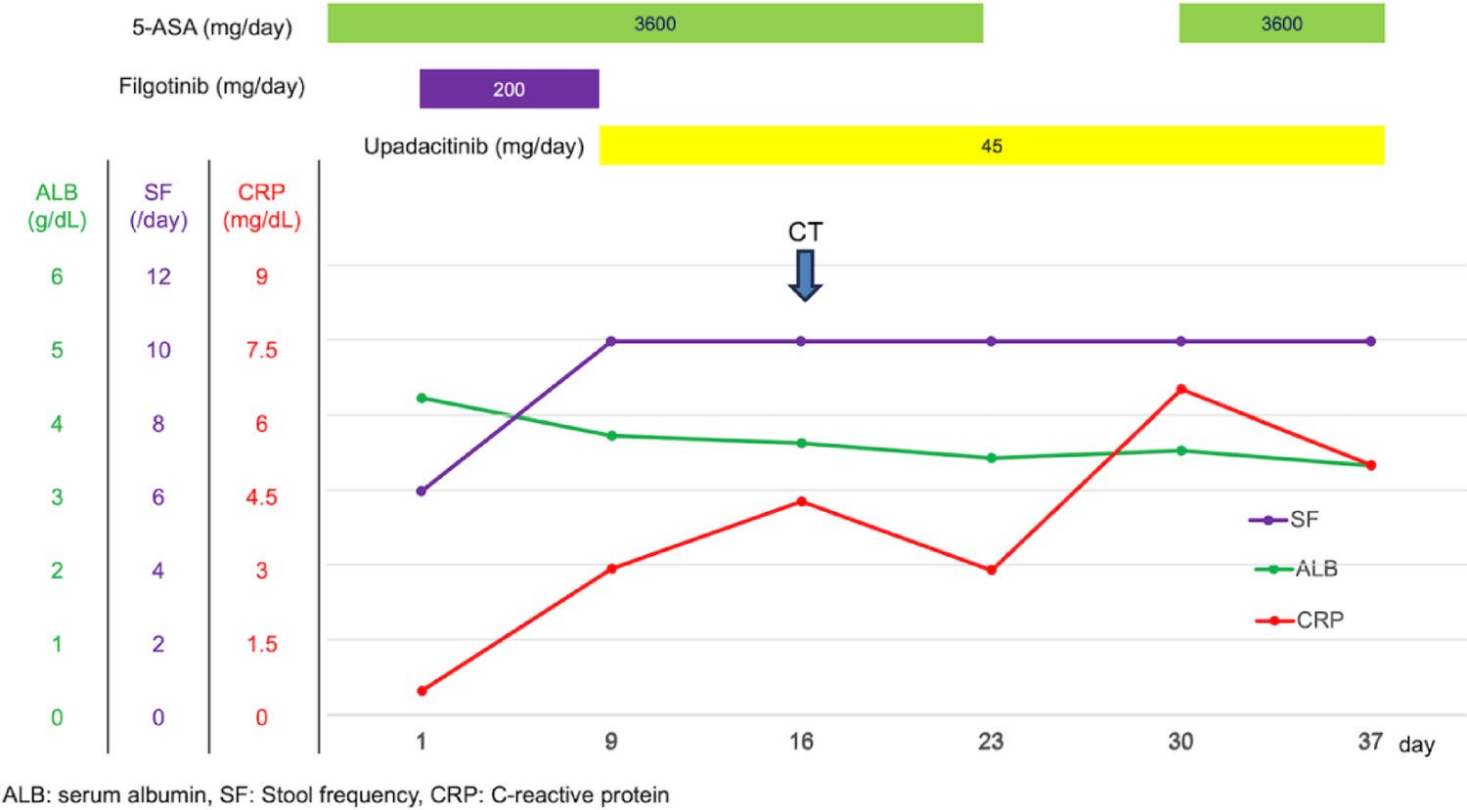
Clinical course after relapse. Nine days after filgotinib initiation, the symptoms persisted, and her C-reactive protein (CRP) levels increased. Filgotinib is switched to Upadacitinib. However, there was no enhancement in either symptoms or CRP levels. A suspected adverse reaction to the increased dose of 5-aminosalicylic acid facilitated its temporary discontinuation, but the patient’s condition did not enhance

Two months later, while on 5-ASA maintenance, she experienced moderate relapse with six bowel movements per day and mild hematochezia. At that time, her C-reactive protein (CRP) level was 0.47 mg/dL (Table S1). Sigmoidoscopy revealed continuous, diffusely distributed fine-granular mucosa with scattered erosions (Fig. 1). To induce remission, filgotinib (200 mg/day) was initiated, and the subsequent clinical course is illustrated in Fig. 2.

**Fig. 3:**
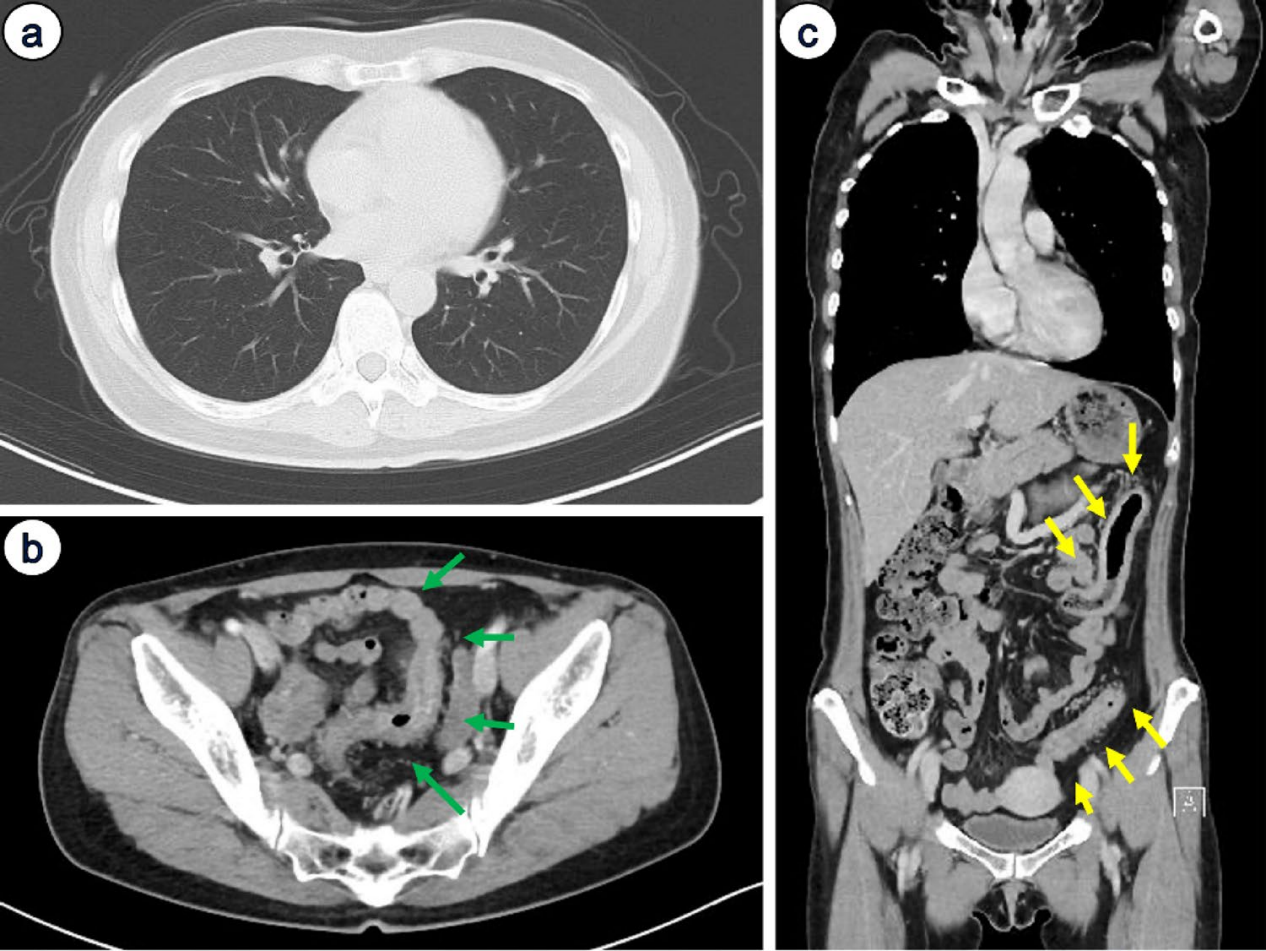
Computed tomography (CT) findings after upadacitinib initiation. **a**–**c** Contrast-enhanced CT revealed wall thickening of the left colon, with no abnormal findings in other organs (**a** axial image at the level of the lungs, **b** axial image at the level of the sigmoid colon, and **c** coronal image). Green arrows indicate the sigmoid colon; yellow arrows indicate the left transverse and sigmoid colon

Nine days after starting filgotinib, the patient experienced worsening diarrhea (approximately 10 bowel movements/day) and increased CRP levels. Consequently, filgotinib was switched to upadacitinib (45 mg/day). However, her symptoms persisted, and CRP levels increased to 6.51 mg/dL. Serum albumin levels remained mildly reduced. On day 7 of upadacitinib administration, contrast-enhanced computed tomography (CT) revealed colonic wall thickening from the rectum to the transverse colon, and no other cause for the increased CRP was identified (Fig. 3). Because of suspected intolerance to high-dose 5-ASA, the medication was discontinued for one week; however, no clinical enhancement was observed.

**Fig. 4:**
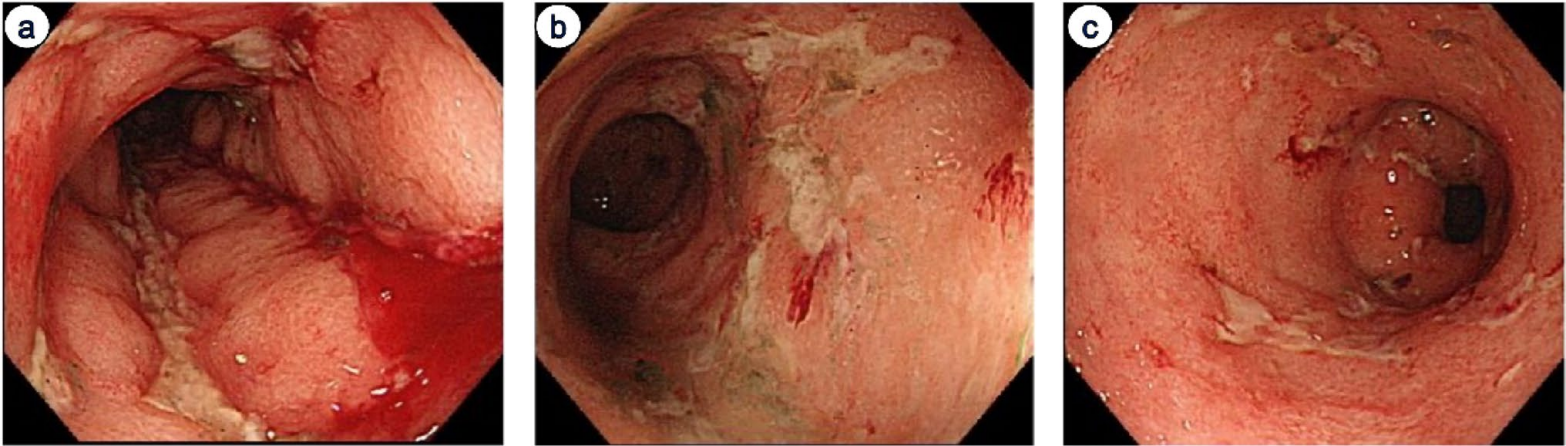
Colonoscopy on admission. **a**–**c** Colonoscopy revealed the loss of vascular pattern, multiple shallow ulcers and longitudinal ulcers (**a** descending colon, **b** sigmoid colon, and **c** rectum)

Upadacitinib was deemed ineffective, and the patient was admitted 33 days after its initiation for tacrolimus therapy. On admission, the CRP and serum albumin levels were 1.63 mg/dL and 3.6 g/dL, respectively (Table 1). Stool culture revealed no pathogenic bacteria, and the cytomegalovirus antigen test was negative. Colonoscopy performed upon admission revealed a bleeding longitudinal ulcer in the descending colon and erosions in the sigmoid colon and rectum (Fig. 4).

**Table 1:** Laboratory findings on admission

WBC	7450 /µL	AST	15 IU/L	K	4.1 mEq/L
Seg	64.9%	ALT	12 IU/L	Cl	101 mEq/L
Eosino	1.1%	LDH	157 IU/L	BS	87 mEq/L
Baso	0.7%	ALP	207 IU/L	CK	23 IU/L
Lymph	27.0%	γ-GTP	22 IU/L	CRP	1.63 mg/dL
Mono	5.8%	T-Bil	0.3 g/dL	CMV Ag	Negative
RBC	395 × 10^4^/µl	Alb	3.6 mg/dL	*Clostridium*	Negative
Hb	11.6 g/dl	BUN	12 mg/dL	*difficile* toxin	
Ht	36.1%	Cr	0.64 mg/dL		
Plt	460 × 10^3^/µL	Na	140 mEq/L		

WBC: white blood cell, Seg: segmented neutrophils, Eosino: eosinophils, Baso: basophil, Lymph: lymphocytes, Mono: monocytes, RBC: red blood cell, Hb: hemoglobin, Ht: hematocrit, Plt: platelets, AST: aspartate aminotransferase, ALT: alanine aminotransferase, LDH: lactate dehydrogenase, ALP: alkaline phosphatase, γ-GTP: γ-glutamyl transferase, T-bil: total bilirubin, Alb: albumin, BUN: blood urea nitrogen, Cr: creatinine, Na: sodium, K: potassium, Cl: chloride, BS: blood sugar, CK: creatin kinase, CRP: C-reactive protein, and CMV: cytomegalovirus

**Fig. 5:**
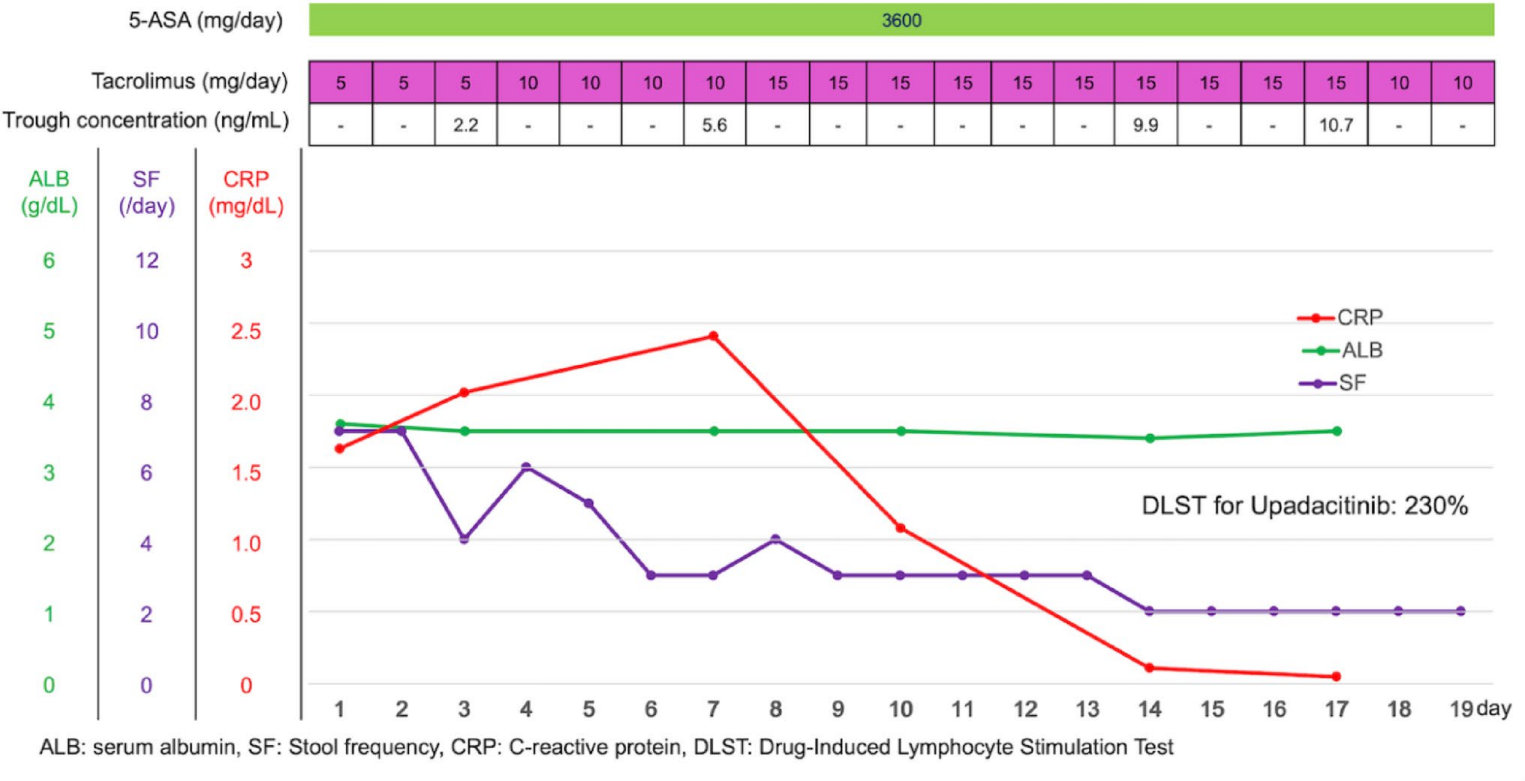
Clinical course after admission. Upon admission, upadacitinib is discontinued, and tacrolimus is initiated at 0.1 mg/kg, with dose adjustments guided by blood trough levels. The patient’s diarrhea gradually improved, and CRP levels decreased. Clinical remission is achieved by day 10 of hospitalization. A drug-induced lymphocyte stimulation test is positive for upadacitinib. The patient is discharged on day 19

**Fig. 6:**
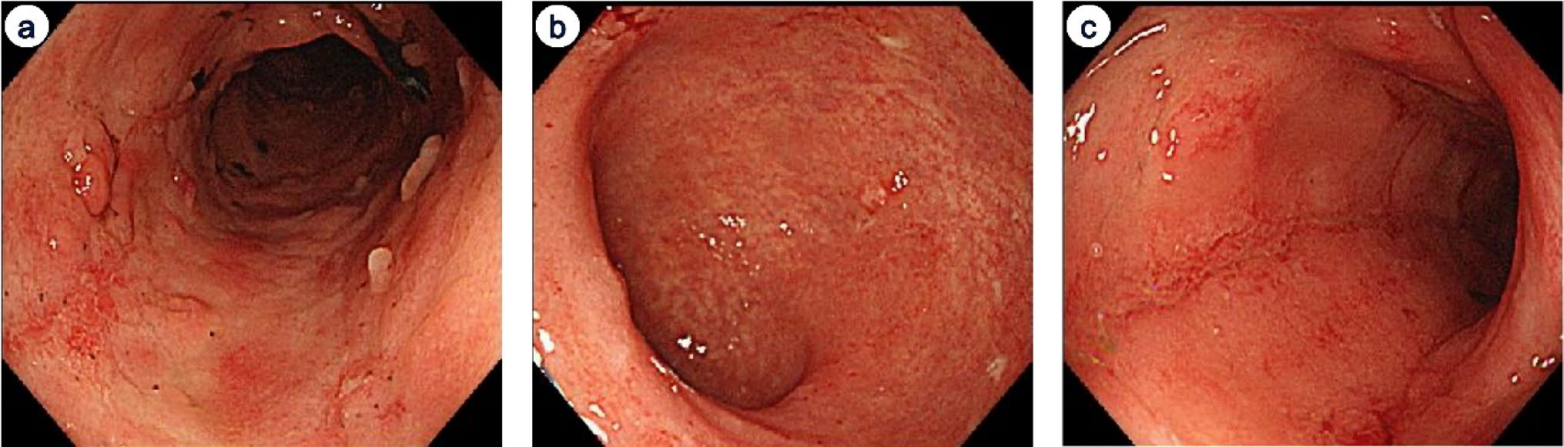
Colonoscopy performed 14 days after admission. **a**–**c** Colonoscopy demonstrated resolution of the ulcers, with scattered small erosions remaining (**a** descending colon, **b** sigmoid colon, and **c** rectum)

The clinical course during hospitalization is illustrated in Fig. 5. On day 1 of hospitalization, upadacitinib was discontinued and tacrolimus was initiated at 5 mg/day (0.1 mg/kg), with dose adjustments guided by trough levels. The patient’s diarrhea gradually improved, and CRP levels decreased. Clinical remission was achieved on day 10. On day 14, colonoscopy revealed the resolution of longitudinal ulcers and erosions (Fig. 6).

A drug-induced lymphocyte stimulation test (DLST) was positive for upadacitinib, with a stimulation index of 230%. Considering the worsening of symptoms during upadacitinib treatment, improvement after its discontinuation, and the positive DLST result, the clinical course was considered compatible with a suspected DHR to upadacitinib. The patient was discharged on day 19. After discharge, tacrolimus was switched to azathioprine, and the patient remained in remission for over a year.

## Discussion

DHRs are unpredictable type B adverse drug reactions that occur in predisposed individuals. Diagnostic assessment involves patient history, skin testing, in vitro assays, and provocation tests, considering the clinical context and available diagnostic options [9]. In this case, diarrhea persisted after initiating JAK inhibitors, and the CRP levels increased. Recognition of potential drug hypersensitivity was challenging because the clinical manifestations closely resembled a flare of ulcerative colitis. The patient did not exhibit typical systemic allergic features such as rash or eosinophilia, and the elevated CRP levels with endoscopic worsening were compatible with primary non-response to JAK inhibitor therapy. Given that JAK inhibitors may require several weeks to achieve maximal clinical efficacy, delayed therapeutic response was initially considered. Therefore, distinguishing between drug-related immune reactions and treatment non-response in the early phase of JAK inhibitor therapy can be clinically difficult. However, the relatively stable serum albumin levels despite marked CRP elevation were somewhat atypical for severe UC exacerbation. Following withdrawal of upadacitinib, the patient’s symptoms and inflammatory markers gradually improved. In addition, the DLST for upadacitinib was positive. Although the DLST has limited sensitivity and its specificity varies depending on the drug and clinical context, it provided supportive evidence in this case [10]. Based on the temporal relationship between drug exposure and clinical deterioration, subsequent improvement after drug withdrawal with immunomodulatory therapy, and DLST positivity, the clinical course was considered compatible with suspected drug hypersensitivity to upadacitinib.

DHRs can manifest as cutaneous eruptions (morbilliform drug eruptions, urticaria, angioedema, and fixed drug eruptions), multi-organ involvement, vasculitis, or fever. Various mechanisms have been proposed to explain how low-molecular-weight drugs elicit immune responses, including the hapten hypothesis, pharmacological interaction (p-i) hypothesis, direct mast cell activation through Mas-related G protein-coupled receptor member X2, and altered peptide repertoire model, in which drug–human leukocyte antigen interactions modify peptide presentation and trigger T-cell responses [11]. In this case, colonic injury occurred without associated skin eruptions or fever. The successful use of tofacitinib in drug-induced hypersensitivity syndrome/drug reactions with eosinophilia demonstrates the JAK–STAT signaling pathway as a potential therapeutic target, as revealed by single-cell RNA sequencing of skin and blood samples from refractory patients [12]. Transcriptomic and immunological analyses of prolonged drug reaction with eosinophilia and systemic symptoms (DRESS) revealed the upregulation of C–C chemokine receptor type 8 (CCR8) and C–X–C chemokine receptor type 3 (CXCR3) pathways, along with the expansion of CCR8⁺ T helper type 2 and CXCR3⁺ effector memory T cells. Notably, both in vitro inhibition assays and clinical observations have demonstrated that JAK inhibitors, including tofacitinib and upadacitinib, effectively suppresses these pathogenic responses [13]. Additionally, a case report described a patient with drug-induced hypersensitivity syndrome and cardiac involvement who was successfully treated with the JAK1/3 inhibitor tofacitinib, indicating that JAK inhibition may represent a promising therapeutic approach for severe or refractory cases [14]. In summary, these findings indicate that upadacitinib may have masked certain manifestations of drug-induced hypersensitivity in this case.

To the best of our knowledge, no cases of upadacitinib-induced drug hypersensitivity have been reported in patients with UC. However, severe hypersensitivity has been described with other JAK inhibitors—cases of DRESS syndrome have occurred in patients with systemic lupus erythematosus treated with the JAK1 inhibitor GSK2586184 [5], and drug eruptions with morbilliform or exfoliative skin lesions have been reported with the JAK1/2 inhibitor ruxolitinib [6]. Additionally, symmetrical drug-related intertriginous and flexural exanthema and drug-induced urticaria were observed during tofacitinib therapy, indicating that JAK inhibition may, albeit rarely, provoke cutaneous hypersensitivity reactions [7, 8]. Because symptom exacerbation and increased CRP levels were observed after filgotinib initiation, hypersensitivity to filgotinib cannot be completely excluded. However, at that time, the clinical deterioration was considered compatible with primary non-response rather than a drug-related immune reaction, as no systemic allergic manifestations were observed. Additionally, although mesalamine intolerance was suspected, a one-week drug holiday from 5-ASA did not result in clinical improvement, and the patient had previously tolerated 5-ASA therapy without adverse reactions. Therefore, DLST was not performed for filgotinib or 5-ASA. Nevertheless, the absence of confirmatory testing for these agents represents a limitation of this report. Although involvement of shared excipients between upadacitinib and filgotinib is theoretically possible, this remains speculative and unsupported by direct evidence.

In this case, clinical remission and endoscopic improvement were observed after discontinuation of upadacitinib and initiation of tacrolimus. Because symptomatic improvement occurred before therapeutic tacrolimus trough levels were achieved, withdrawal of upadacitinib may have contributed to the early clinical recovery. The efficacy of calcineurin inhibitors in drug allergy has been reported in several studies. In a retrospective cohort study of 19 patients with DRESS syndrome, cyclosporine achieved symptom resolution in 89% of cases, with a mean treatment duration of 5 days and relapse rate of 16% [15]. Oral tacrolimus was effective for gastric lesions caused by prednisolone-induced drug allergy in a patient with UC [16]. Wedel further proposed cyclosporine as a step-up therapy for cases of DRESS that are refractory to systemic corticosteroids [17]. Nevertheless, in routine clinical practice, tacrolimus use for drug hypersensitivity remains off-label, and its efficacy in this context has not been established. In the present case, tacrolimus may have contributed to immune modulation and mucosal healing; however, causality cannot be definitively determined.

In conclusion, this case suggests that drug hypersensitivity to upadacitinib may mimic apparent disease exacerbation in patients with ulcerative colitis. Clinicians should consider the possibility of drug-related immune reactions when clinical deterioration occurs during JAK inhibitor therapy, particularly in the absence of typical allergic manifestations. Although the diagnosis in this case remains presumptive, careful reassessment of the clinical course may facilitate timely recognition and appropriate management.

## Supplementary Information

Below is the link to the electronic supplementary material.

Supplementary Material 1
