# Supplementary material for: Suspected upadacitinib-associated drug hypersensitivity mimicking disease exacerbation in ulcerative colitis
Source: Clin J Gastroenterol. 2026 Apr 27;19(4):841–6. doi: 10.1007/s12328-026-02342-z (PMC13424229; doi:10.1007/s12328-026-02342-z)
Supplement: Supplementary file 1 — Supplementary Material 1 [file 12328_2026_2342_MOESM1_ESM.docx]

**Table caption**

Table S1. Laboratory findings during a moderate relapse while on maintenance therapy with 5-aminosalicylic acid.

| WBC | 6,730 /µL | AST | 15 IU/L | K | 3.8 mEq/L |
| --- | --- | --- | --- | --- | --- |
| Seg | 71.8 % | ALT | 14 IU/L | Cl | 105 mEq/L |
| Eosino | 1.0 % | LDH | 138 IU/L | BS | 90 mg/dL |
| Baso | 0.4 % | ALP | 148 IU/L | CK | 29 IU/L |
| Lymph | 21.0 % | γ-GTP | 16 IU/L | CRP | 0.47 mg/dL |
| Mono | 5.8 % | T-Bil | 0.5 g/dL |  |  |
| RBC | 436 × 10^4^/μl | Alb | 4.2 mg/dL |  |  |
| Hb | 12.7 g/dl | BUN | 9.8 mg/dL |  |  |
| Ht | 39.4 % | Cr | 0.62 mg/dL |  |  |
| Plt | 322 × 10^3^/μL | Na | 141 mEq/L |  |  |

WBC: white bold cell, Seg: segmented neutrophils, Eosino: eosinophils, Baso: basophil, Lymph: lymphocytes, Mono: monocytes, RBC: red blood cell, Hb: hemoglobin, Ht: hematocrit, Plt: platelets, AST: aspartate aminotransferase, ALT: alanine aminotransferase, LDH: lactate dehydrogenase, ALP: alkaline phosphatase, γ-GTP: γ-glutamyl transferase, T-bil: total bilirubin, Alb: albumin, BUN: blood urea nitrogen, Cr: creatinine, Na: sodium, K: potassium, Cl: chloride, BS: blood sugar, CK: creatin kinase, and CRP: C-reactive protein.
